# Research progress on the mechanism of microglia–astrocyte signal crosstalk in central sensitization of vestibular migraine

**DOI:** 10.3389/fnins.2026.1844932

**Published:** 2026-10-23

**Authors:** Tiancan Jin, Weitao Wang, Jinghan Qin, Yaru Liu, Xiaofei Fan, Daopei Zhang, Huailiang Zhang

**Affiliations:** 1 Department of Encephalopathy, The First Affiliated Hospital of Henan University of Traditional Chinese Medicine, Zhengzhou, China; 2 The First Clinical Medical College, Henan University of Traditional Chinese Medicine, Zhengzhou, China; 3 Henan Collaborative Innovation Center for Prevention and Treatment of Major Diseases with Integrated Traditional Chinese and Western Medicine, Zhengzhou, China; 4 Institute of Vertigo Diseases, Henan University of Traditional Chinese Medicine, Zhengzhou, China; 5 Henan Vertigo Disease Diagnosis and Treatment Center, Zhengzhou, China

**Keywords:** astrocyte, central sensitization, microglia, signal crosstalk, vestibular migraine

## Abstract

Vestibular migraine (VM) is a vestibular disorder characterized by recurrent episodes of vertigo, frequently accompanied by headache, nausea, and vomiting, which substantially reduce patients’ quality of life. The pathogenesis of VM remains incompletely understood. Current studies mainly focus on ion channel dysfunction, trigeminovascular mechanisms, and genetic susceptibility. Increasing evidence suggests that neuroinflammation and central sensitization play important roles in the onset and maintenance of VM. Microglia and astrocytes are key mediators of central sensitization, and their bidirectional communication represents an important component of neuroimmune interactions in the central nervous system. Based on recent studies, this review systematically summarizes the interactions and crosstalk between microglia and astrocytes in central sensitization, aiming to provide further insight into the pathophysiology of VM and to explore potential preventive and therapeutic strategies.

## 1 Introduction

Vestibular migraine (VM) is a vestibular disorder characterized by recurrent episodes of vertigo and dizziness, which may be accompanied by nausea, vomiting, and/or headache, and is commonly encountered in clinical practice. A recent nationwide study applying the diagnostic criteria of the *International Classification of Headache Disorders, Third Edition* (ICHD-3), reported that the prevalence of VM in the adult population is approximately 2.7% (Formeister et al., 2018). The clinical presentation of VM is heterogeneous. Patients may exhibit symptoms typical of migraine, such as headache, photophobia, and phonophobia, or may present predominantly with vertigo. In addition, substantial inter-individual variability exists in the clinical manifestations of VM (Furman et al., 2013). Recurrent episodes of vertigo and headache can significantly interfere with daily activities and may reduce work capacity. Some patients also experience emotional disturbances, including anxiety and depression (Ma et al., 2024). Currently, no specific biomarkers are available for VM. Therefore, diagnosis largely relies on clinical history and symptom evaluation, which may further complicate the diagnostic process (Nowaczewska, 2020).

VM is associated with migraine, and most current hypotheses regarding its pathophysiological mechanisms are derived from studies of migraine (Sohn, 2016). Activation of the trigeminovascular system is considered a key mechanism in migraine, contributing to nociceptive signal transmission and triggering neuroinflammatory responses through the release of neuropeptides, such as calcitonin gene-related peptide (CGRP) (Ashina et al., 2019). Concurrently, central sensitization represents another key mechanism involved in migraine chronification and is characterized by heightened responsiveness of the central nervous system (CNS) to sensory stimuli (Suzuki et al., 2022). However, the pathogenesis of VM remains incompletely understood. Current research mainly focuses on several potential mechanisms, including ion channel dysfunction, trigeminovascular activation, and genetic or molecular factors (Baloh, 2020). Recent studies indicate that neuroinflammation and central sensitization may play important roles in the onset and persistence of VM (Zhai et al., 2025b). Central sensitization refers to structural, functional, and neurochemical alterations in the CNS that increase sensitivity to pain and other sensory inputs (Volcheck et al., 2023). Microglia and astrocytes are recognized as key cellular mediators involved in central sensitization (Han et al., 2022). In addition, migraine-related studies have demonstrated that the brain microenvironment, associated peripheral regulatory circuits, and glial cells, including microglia and astrocytes, are essential for maintaining normal brain function (Amani et al., 2023). These observations indicate that microglia and astrocytes may also contribute to the pathogenesis of VM. As understanding of the interactions between the CNS and the immune system has advanced, increasing evidence indicates a close association between these two systems. Neuroinflammation is increasingly regarded as a key factor in the initiation and progression of a range of neurological disorders (Müller et al., 2025). Within the CNS, microglia and astrocytes serve as the primary immunoregulatory cells and are crucial for the maintenance of CNS homeostasis. Microglia function as the resident immune cells of the CNS and play a key role in maintaining brain homeostasis (Sadeghdoust et al., 2024). Astrocytes, the most abundant glial cells in the CNS, are involved in regulating neurotransmission, inflammation, neurogenesis, and synaptic plasticity (Jing et al., 2023). Signaling crosstalk exists between microglia and astrocytes during central neuroinflammatory and immune processes. A deeper understanding of the crosstalk between microglia and astrocytes may help elucidate the molecular mechanisms underlying central sensitization in VM. Furthermore, this line of research may offer a theoretical foundation for developing novel targeted therapeutic strategies. Therefore, based on recent evidence, this review summarizes current progress in understanding how microglia–astrocyte crosstalk promotes central sensitization in VM and aims to identify potential therapeutic targets for VM prevention and treatment.

## 2 Microglia-astrocyte crosstalk contributes to central sensitization in VM

### 2.1 Modes and signaling molecules involved in microglia–astrocyte interactions and crosstalk

Microglia and astrocytes interact to preserve physiological homeostasis within the healthy CNS. Alterations in the microenvironment can modify their interaction patterns, triggering specific cellular responses that may contribute to pathological processes (Sun et al., 2023). Growing evidence suggests that these interactions are involved in the pathogenesis of disorders including Alzheimer’s disease, Parkinson’s disease, multiple sclerosis, and HIV-associated neuropathy (Jha et al., 2019). Previous studies show that activated microglia can polarize into M1 (pro-inflammatory) or M2 (anti-inflammatory) types, each with distinct roles in inflammation (Atta et al., 2023). Similarly, reactive astrocytes have been classified into A1 (pro-inflammatory) and A2 (anti-inflammatory) phenotypes, which are generally linked to neurotoxic and neuroprotective functions, respectively (Liddelow et al., 2017). However, growing evidence indicates that microglial and astrocytic responses cannot be adequately described by simple binary classification schemes. Instead, they are shaped by multiple factors, including the type of stimulus and the stage of disease, resulting in dynamic and heterogeneous cellular states (Paolicelli et al., 2022). It is therefore more appropriate to describe these responses according to multiple dimensions, including the type of stimulus, intercellular signaling molecules, intracellular signaling pathways, and functional outcomes. Furthermore, there is currently no universally accepted definition of terms such as “interaction” and “crosstalk” in the existing literature. In this review, “interaction” is used as a broad term to describe functional relationships between different cell populations. In contrast, “crosstalk” specifically refers to experimentally supported, signal-mediated communication between microglia and astrocytes. To clarify the criteria for applying this term, we define the minimum evidence required for microglia–astrocyte crosstalk as experimental evidence that a signal produced or regulated by one cell type can influence the molecular or functional response of the other. Both microglia and astrocytes should be clearly identified. There should also be experimental evidence supporting a signal-mediated connection between the two cell types. Such evidence may include a temporal or functional association between a signal from one cell type and a response in the other, a ligand–receptor relationship, or changes in downstream signaling. Pharmacological or genetic manipulation can provide further support when appropriate. Bidirectional communication is not required to establish crosstalk. When the available evidence supports signaling in only one direction, we explicitly indicate the direction without implying bidirectional or reciprocal communication.

Experimental studies using lipopolysaccharide (LPS) stimulation have shown that cytokine induction in glial cells occurs in a sequential manner. Pro-inflammatory cytokine expression in microglia peaks 2–4 h post-LPS, while cytokine and chemokine induction in astrocytes is relatively delayed, reaching its peak at 12 h (Norden et al., 2016). This suggests that, in certain acute inflammatory environments, microglia may detect stimuli at an early stage and influence the subsequent astrocyte response by secreting signaling molecules. Wolfbeisz et al. (2025) further showed that microglia-derived TNF-α mediates inflammatory signaling to astrocytes and promotes their response. This process involves nuclear factor kappa B (NF-κB) translocation to the nucleus in astrocytes (Wolfbeisz et al., 2025). Furthermore, Liddelow et al. (2017) found that reactive microglia release IL-1α, TNF-α, and C1q, which induce molecular and functional changes in astrocytes. Another study showed that conditioned medium from LPS-stimulated microglia induces NLRP3 inflammasome activation and pro-inflammatory cytokine release in astrocytes, suggesting that microglia-derived soluble factors can regulate inflammatory signaling in astrocytes (Santos-García et al., 2025). In a migraine model induced by dural infusion of inflammatory soup (IS), microglia-derived Interleukin-18 (IL-18) was shown to act on IL-18 receptor (IL-18R) in astrocytes (Gong et al., 2020). These studies suggest that, under specific pathological conditions, reactive microglia can regulate astrocytes through multiple pathways.

Furthermore, reactive astrocytes can also regulate microglial responses through multiple signaling pathways. In response to specific signals, reactive astrocytes can release inflammatory mediators, reactive oxygen species, and glutamate, and may also impair blood–brain barrier (BBB) function (Sofroniew and Vinters, 2010). Microglia-derived TNF-α, C1q, and other signaling molecules can promote astrocytic responses and the release of inflammatory mediators, including IL-1β, TNF-α, and nitric oxide (NO) (Kwon and Koh, 2020; Lawrence et al., 2023). These inflammatory mediators, including TNF-α and IL-1β, in turn enhance inflammatory responses in microglia and promote the production of additional inflammatory mediators (Hoogland et al., 2015; Liu et al., 2022). These findings suggest that reciprocal signaling between microglia and astrocytes may amplify inflammatory responses. Chemokines may also mediate signaling between astrocytes and microglia. Studies demonstrate that TNF-α-induced astrocytes can express and release the chemokine C-C motif ligand 2 (CCL2). CCL2 can subsequently modulate microglial inflammatory responses, while inhibition of its receptor CCR2 attenuates this effect (He et al., 2016). This *in vitro* experiment demonstrates that astrocyte-derived CCL2 acts on microglia via CCR2, supporting directional signaling from astrocytes to microglia. In a rodent model of chronic ischaemia-induced pain, astrocyte-derived CSF1 acts on CSF1R expressed by microglia, promoting microglial responses and BDNF production and subsequently increasing the excitability of spinal dorsal horn neurons. Inhibition of this pathway attenuates microglial responses, central sensitization, and allodynia. This study provides relatively direct evidence for astrocyte-to-microglia signaling (Tang et al., 2018). Overall, current evidence indicates that microglia and astrocytes can communicate through cytokines, chemokines, and ligand–receptor interactions under specific pathological conditions. The strength, timing, and duration of these signals may vary with the pathological context and influence the reactive states of both cell types.

### 2.2 Specific mechanisms of glial cell crosstalk contribute to central sensitization in VM

#### 2.2.1 Glial inflammatory mediators contribute to central sensitization

Increasing evidence suggests that central sensitization is driven by neuroinflammation, which involves leukocyte infiltration, glial activation, and elevated levels of inflammatory mediators (Han et al., 2022). Song et al. (2025) found that in VM animal models, activation of the C-X-C motif chemokine ligand 10 (CXCL10) / C-X-C motif chemokine receptor 3 (CXCR3) signaling pathway through the phosphatidylinositol 3-kinase (PI3K) / protein kinase B (AKT) pathway was accompanied with increased expression of pro-inflammatory cytokines, including IL-1β, IL-6, and TNF-α, in regions related to the trigeminal nucleus caudalis (TNC). The present findings indicate that inflammation-related pathways are involved in the development and progression of VM (Song et al., 2025). The spinal trigeminal nucleus caudalis (Sp5C), also termed TNC and referred to as Vc in some studies, is hereafter referred to as TNC. An animal study showed that IL-1β levels were significantly increased in both the vestibular nucleus (VN) and TNC in a mouse model of VM, suggesting a possible role of neuroinflammation in VM-related central sensitization (Zhai et al., 2025b). Zhai et al. (2025a) observed reactive changes in glial cells in the VN -TNC region in VM mouse models. Stimulated microglia secrete inflammatory mediators, including TNF-α, IL-1β, and IL-6, thereby promoting central sensitization (Chen et al., 2018). Specifically, IL-1β and TNF-α may promote central sensitization by enhancing excitatory synaptic transmission, reducing inhibitory signaling, and activating cyclooxygenase-2 (COX-2) (Samad et al., 2001). Additionally, reactive microglia upregulate P2X4 receptor (P2X4R), promote the synthesis and release of brain-derived neurotrophic factor (BDNF), and impair the function of the cotransporter KCC2 through the BDNF–TrkB signaling pathway, thereby altering Cl^−^ homeostasis and driving central sensitization (Ferrini and De Koninck, 2013). Astrocyte-derived chemokines also participate in this process. For example, CCL2 produced by astrocytes activates the extracellular signal-regulated kinase (ERK) pathway in neurons of the dorsal horn through its receptor CCR2, thereby enhancing N-methyl-D-aspartate (NMDA) receptor activity, increasing neuronal excitability, and promoting central sensitization. Another astrocyte-derived chemokine, C-X-C motif chemokine ligand 1 (CXCL1), activates ERK and cAMP response element-binding protein (CREB) signaling in spinal neurons through its receptor CXCR2, thereby facilitating synaptic plasticity and maintaining the central sensitized state (Zhang et al., 2013; Ji et al., 2018). Therefore, reactive microglia and astrocytes promote the production of inflammatory mediators that promote central sensitization. These mediators can further act on microglia and astrocytes, thereby contributing to the development of VM mediated by central sensitization.

#### 2.2.2 Glial interactions and Trigeminovascular system sensitization

Abnormalities in the trigeminal neurovascular pathway are one of the key mechanisms underlying VM. The trigeminal ganglion (TG), which contains the sensory neuron cell bodies transmitting afferent signals from the craniofacial region and meninges, is a fundamental component of the trigeminovascular system (Zhang et al., 2023a). When the trigeminal ganglion and its fibers are activated, vasoactive neuropeptides such as CGRP are secreted. This release triggers a cascade of events that promote vasodilation and increased vascular permeability, resulting in plasma protein extravasation, sterile inflammation, and activation of nociceptive receptors in the dural and meningeal vasculature. Pain signals are subsequently transmitted to the CNS via trigeminal afferents, thereby contributing to migraine-like pain (Moskowitz et al., 1979). Activation of the TNC is an important feature of central sensitization, and increased neuronal excitability in this region is closely associated with the progression of central sensitization (Lu et al., 2024). Evidence suggests that activation of the TNC significantly elevates CGRP levels in both local and trigeminal ganglion compartments (Christiansen et al., 2025). Moreover, Zhang et al. (2023b) reported that glutamatergic projections from the TNC to the VN mediate vestibular dysfunction in a chronic nitroglycerin (NTG)-induced rat model. Suppressing glutamatergic TNC neurons markedly attenuates NTG-induced vestibular dysfunction (Zhang et al., 2023b). Taken together, these findings indicate a potential mechanistic relationship between the trigeminovascular system and VM-related symptoms.

Glial cells are also critically involved in this process. Previous studies have shown reactive changes in microglia and astrocytes within the trigeminal system in migraine-related conditions. Zhang et al. (2022) reported that repeated administration of IS induced reactive changes in astrocytes and microglia within the TNC in mice, and that the duration of glial reactivity was positively correlated with trigeminovascular sensitization. This suggests that both astrocytes and microglia may be involved in trigeminovascular sensitization. Another study showed that, in a chronic migraine (CM) model induced by repeated NTG administration, P2Y12 receptor expression was increased in microglia within the TNC and regulated morphological changes and inflammatory cytokine production in these microglia. Inhibition of the related pathway attenuated microglial reactivity and pain hypersensitivity (Jing et al., 2019). In a triptan-induced medication-overuse headache (MOH) model, Wang et al. (2026) observed persistent astrocyte reactivity in the TNC. Chemogenetic inhibition of TNC astrocytes prevented the development of long-term cutaneous allodynia and latent sensitization, supporting the involvement of astrocytes in trigeminovascular sensitization (Wang et al., 2026). These findings collectively suggest that microglia and astrocytes are involved in migraine-associated trigeminovascular sensitization.

Building on these findings, several studies have further shown functional interactions between glial cells and TNC neurons. Studies have shown that in CM mice, stimulation of microglial P2X4Rs increases BDNF expression and leads to BDNF release, thereby mediating neuronal excitability in the TNC. This effect is reflected by increased levels of phosphorylated extracellular signal-regulated kinase (p-ERK) and CGRP (Long et al., 2020). Repeated NTG administration also induces NLRP3 inflammasome activation and increases NLRP3 expression in TNC microglia, subsequently activating the IL-1β signaling pathway. Inhibition of NLRP3 and IL-1β alleviates hyperalgesia and suppresses the elevation of biomarkers associated with central sensitization, including p-ERK, c-Fos, and CGRP in the TNC. These effects may be related to microglia–neuron signaling pathways (He et al., 2019). Zhou et al. (2020) found that excitatory amino acid transporter 2 (EAAT2) was mainly expressed in astrocytes in the TNC. In rats with CM, EAAT2 expression in the TNC was reduced and was accompanied by impaired glutamate homeostasis. Upregulation of EAAT2 reduced NMDA receptor-related signaling and abnormal neuronal synaptic plasticity, thereby attenuating central sensitization. These findings suggest that astrocytes may regulate TNC neuronal excitability through EAAT2-mediated glutamate clearance (Zhou et al., 2020). Additionally, the advent of optogenetics has accelerated research on neuropathic pain mechanisms, allowing precise activation or inhibition of specific cells within neural circuits (Li et al., 2022). Furthermore, optogenetic stimulation of uniquely phenotypic glial cells, such as microglia and astrocytes, has been shown to have therapeutic effects on neurodegenerative diseases (Eldar et al., 2026). Kc et al. (2023) found that optogenetic inhibition of TNC astrocytes reversed hyperalgesia and anxiety-like behaviors in a rat model of trigeminal neuralgia. These studies suggest that glial cells may contribute to trigeminovascular sensitization by regulating the excitability of TNC neurons.

Compared with evidence for glial reactivity and glia–neuron interactions, direct experimental evidence for microglia–astrocyte crosstalk in the trigeminovascular system in migraine or VM remains limited. Gong et al. (2020) found that, in a migraine model induced by dural administration of IS, IL-18 was mainly localized in microglia, whereas IL-18R was mainly expressed in astrocytes. Blocking IL-18-related signaling attenuated astrocyte reactivity and migraine-like pain hypersensitivity (Gong et al., 2020). These findings provide relatively direct cell-specific evidence that microglia transmit inflammatory signals to astrocytes. Evidence from a trigeminal neuropathic pain model also provides mechanistic support. Microglia-derived C1q promotes astrocyte reactivity in the TNC and further increases the excitability of nociceptive neurons, suggesting that interglial signaling may influence trigeminal neuronal excitability (Asano et al., 2020). Furthermore, Hu et al. (2025) performed single-nucleus RNA sequencing (snRNA-seq) of the TNC in an NTG-induced rat model of CM and found marked state remodeling in both microglia and astrocytes. Further intercellular communication analysis revealed an abnormal communication network between microglia and astrocytes under CM conditions (Hu et al., 2025). In summary, existing evidence supports possible unidirectional or bidirectional regulation between microglia and astrocytes under pathological conditions. Such crosstalk may contribute to sustained trigeminovascular sensitization by altering the local inflammatory microenvironment in the TNC and neuronal excitability.

#### 2.2.3 Glial interaction–mediated neurotransmitter imbalance

Dysregulation of neurotransmitter metabolism is a key biochemical feature of central sensitization, and glutamatergic dysfunction appears to contribute to the pathogenesis of VM. Previous studies have shown that glutamatergic neurons projecting from the TNC to the VN mediate vestibular dysfunction in a chronic NTG-induced rat model. Silencing these glutamatergic TNC-VN projection neurons significantly improves vestibular dysfunction in rats (Zhang et al., 2023b). This suggests that excessive activation of the glutamatergic pathway may be an important mechanism underlying vestibular symptoms in migraine. In CM mouse models, a marked decrease in energy metabolism and an increase in excitatory neurotransmitters such as glutamate are observed during central sensitization (Gao et al., 2024). This finding indicates that glutamate may also mediate migraine symptoms by participating in the central sensitization process. Excessive glutamate levels induce postsynaptic membrane depolarization, relieving the magnesium block of NMDA receptor channels and allowing glutamate binding and inward cation currents. Activation of NMDA receptors permits Ca^2+^ influx, triggering multiple intracellular signaling pathways that sustain central sensitization (Latremoliere and Woolf, 2009). Glial cells also contribute to glutamate dysregulation. When microglia exhibit reactive changes, in addition to releasing the aforementioned inflammatory mediators, they may also release glutamate via vesicular exocytosis (Illes et al., 2020), leading to an increase in glutamate concentration. Microglia can also directly stimulate astrocyte P2Y1 receptor-mediated signaling by releasing adenosine triphosphate (ATP), which also results in astrocyte glutamate release (Nobili et al., 2022). Additionally, microglia can enhance inflammatory responses in astrocytes through activation of the NF-κB signaling pathway (Gao et al., 2023). Studies have demonstrated that reactive changes in astrocytes affect glutamate uptake (Putatunda et al., 2014). Moreover, astrocytes are essential for maintaining glutamate homeostasis through glutamate transporters and glutamine synthetase. However, microglial reactivity can disrupt this regulatory system, resulting in elevated glutamate concentrations in the synaptic cleft. Inhibition of microglial activation has been shown to restore the glutamate uptake capacity of astrocytes (Guan et al., 2022).

The glutamate-glutamine cycle (GGC) is a critical metabolic pathway in the CNS that maintains glutamate homeostasis and prevents excitotoxic neuronal damage through metabolic coupling between astrocytes and neurons (Liu et al., 2026). Elevated levels of the glutamate–glutamine complex (Glx) in the bilateral thalamus during VM attacks suggest that disruption of this cycle may contribute to the pathogenesis of VM (Wang et al., 2024). In addition, Microglia can impair synaptic glutamate clearance by pruning astrocytic processes, thereby reducing astrocytic coverage of vasopressin neurons and promoting activation of extrasynaptic NMDA receptors, which enhances neuronal excitability (Gu et al., 2025). Excessive glutamate accumulation can further lead to NMDA receptor–mediated neuronal dysfunction (Romanos et al., 2020). Furthermore, neuronal hyperactivation and damage can trigger excessive activation of the trigeminovascular system, thereby exacerbating headache symptoms (Gao et al., 2024). Collectively, these findings suggest that abnormal microglia–astrocyte interactions disrupt glutamate homeostasis, thereby facilitating central sensitization and neuronal dysfunction and contributing to the clinical manifestations of VM.

## 3 Key glial signaling pathways involved in central sensitization in VM

### 3.1 Intracellular signaling pathways in glial cells

Glial responses to noxious stimuli depend on various membrane receptors, ion channels, and intracellular signaling pathways. In migraine research, the strongest mechanistic evidence to date has focused mainly on microglia. In CM models, microglia can activate the p38 mitogen-activated protein kinase (p38 MAPK) signaling pathway through P2Y13 receptor (P2Y13R). Inhibition of either P2Y13R or p38 MAPK attenuates microglial reactivity and reduces pain sensitization in CM mice (Yang et al., 2025). In addition to releasing inflammatory mediators, microglia can also influence neuronal function through neurotrophic factors. Long et al. (2020) found that P2X4R signaling was associated with p38 MAPK phosphorylation and increased BDNF expression in a CM animal model and in BV2 microglia. Further experiments in BV2 cells showed that ATP-induced P2X4R stimulation promoted p38 MAPK phosphorylation and the synthesis and release of BDNF. Blocking P2X4R or inhibiting p38 MAPK attenuated these effects, suggesting that the P2X4R–p38 MAPK–BDNF pathway may represent an important signaling axis in microglia (Long et al., 2020). Furthermore, microglia may regulate central sensitization through the triggering receptor expressed on myeloid cells 2 (TREM2)–spleen tyrosine kinase (SYK) signaling cascade. TREM2 interacts with its adaptor protein DNAX-activating protein 12 (DAP12) to modulate SYK signaling (Sun et al., 2020), and activation of SYK subsequently promotes downstream NLRP3 inflammasome activation (Mócsai et al., 2010). Fan et al. (2024) reported that metformin alleviates central sensitization in CM by modulating microglial reactivity and NLRP3 inflammasome activity via the TREM2–SYK pathway. Zhai et al. (2025b) found that transient receptor potential vanilloid 2 (TRPV2) expression was increased in TNC microglia in a mouse model of VM, accompanied by enhanced NLRP3 inflammasome activity and increased levels of proinflammatory mediators. Further *in vitro* experiments in BV2 cells confirmed functional TRPV2 activity and showed that TRPV2 inhibition reduced NLRP3 inflammasome activation. These findings suggest that the TRPV2–Ca^2+^–NLRP3 pathway may represent an important signaling axis in microglia in VM (Zhai et al., 2025b). However, direct evidence on intracellular signaling in astrocytes remains limited in migraine research. In a CM model induced by repeated NTG administration, Tang et al. (2024) found that Xiongzhi Dilong decoction (XZDL) increased α7 nicotinic acetylcholine receptor (α7nAChR) expression and its colocalization with glial fibrillary acidic protein (GFAP) in the TNC. XZDL also reduced GFAP expression and suppressed JAK2/STAT3/NF-κB signaling. Blocking α7nAChR reversed these effects, suggesting that α7nAChR-related JAK2/STAT3/NF-κB signaling is involved in astrocyte reactivity and central sensitization in CM (Tang et al., 2024). Another study showed that cortical spreading depolarization (CSD) induces neuronal release of high-mobility group box 1 (HMGB1), mainly within small extracellular vesicles (sEVs), which are subsequently taken up by surrounding astrocyte processes and promote NF-κB activation (Kaya et al., 2023). Widespread astrocytic NF-κB nuclear translocation after CSD has also been observed in a mouse model of familial hemiplegic migraine type 1 (FHM1) (Dehghani et al., 2021). These findings suggest that HMGB1–NF-κB signaling may contribute to migraine-related cortical neuroinflammation.

### 3.2 Bidirectional signaling between microglia and astrocytes

Microglia and astrocytes can form a bidirectional signaling network via cytokines, chemokines, complement and other soluble mediators, which in turn modulates neuroinflammation and neuronal excitability. Among these, the IL-18/IL-18R pathway represents a relatively well-supported microglia–astrocyte signaling axis. Gong et al. (2020) found in an experimental migraine model that toll-like receptor 4 (TLR4) signaling in microglia promotes IL-18 production. IL-18 was mainly localized in microglia, whereas IL-18R was mainly expressed in astrocytes. Blocking IL-18 signaling reduced NF-κB activation and astrocyte reactivity and attenuated migraine-like pain hypersensitivity. These findings suggest that the microglial TLR4–IL-18–astrocytic IL-18R pathway may mediate inflammatory signaling from microglia to astrocytes in migraine (Gong et al., 2020). Furthermore, Liddelow et al. (2017) showed in cell culture and *in vivo* experiments that microglia-derived IL-1α, TNF, and C1q can induce specific reactive changes in astrocytes. However, this signaling mechanism has been primarily demonstrated in other neuroinflammatory disease models. Previous studies have shown that TNF-α-stimulated astrocytes can express and release CCL2, which regulates inflammatory responses in microglia through CCR2 (He et al., 2016). Consistent with this mechanism, a study in rats with CM showed that increased CCL2 expression in the TNC was mainly colocalized with astrocytes, whereas CCR2 was associated with microglia. Recombinant CCL2 enhanced NLRP3-related inflammatory responses in microglia and increased central sensitization-related markers, including c-Fos and p-ERK, whereas inhibition of CCL2 or NLRP3 partially reversed these changes (Wang et al., 2025). Together, these findings support CCL2–CCR2 signaling as a potential mechanism of astrocyte-to-microglia communication in CM, with NLRP3 acting as a downstream inflammatory pathway in microglia. Studies of neuropathic pain have shown that increased astrocyte-derived C-X-C motif ligand 12 (CXCL12) in the spinal dorsal horn acts on C-X-C chemokine receptor type 4 (CXCR4) in microglia. Blocking CXCR4 or inhibiting microglial responses attenuates CXCL12-induced mechanical allodynia and reduces the levels of inflammatory mediators, including TNF-α, IL-1β, and IL-6 (Luo et al., 2016). These findings suggest that the CXCL12–CXCR4 axis may mediate signaling from astrocytes to microglia.

### 3.3 Downstream effects of glial signaling networks

Existing evidence suggests that, although different glial signaling pathways have distinct upstream mechanisms, their downstream effects are critical for linking these pathways to central sensitization. Firstly, multiple intracellular signaling pathways in microglia can promote central sensitization by increasing inflammatory output. The MAPK pathway regulates the synthesis and secretion of inflammatory cytokines in microglia in response to various stressors (Bachstetter et al., 2011). Studies in CM models have shown that P2Y13R–p38 MAPK signaling increases microglial reactivity and the levels of inflammatory mediators, including TNF-α and IL-1β, accompanied by increased CGRP and c-Fos expression in the TNC. Further studies have shown that microglia-derived IL-1β in the TNC acts on neuronal IL-1R, thereby contributing to central sensitization in migraine. Blocking this signaling pathway reduces the expression of p-ERK, c-Fos, and CGRP and alleviates allodynia (He et al., 2019). Furthermore, CGRP has been demonstrated to play a key role in the central sensitization process of migraine (Iyengar et al., 2019). Similarly, TREM2–SYK–NLRP3 and TRPV2–Ca^2+^–NLRP3 signaling are associated with increased levels of inflammatory mediators (Fan et al., 2024; Zhai et al., 2025b). This suggests that inflammatory mediator release may be a common downstream mechanism through which different microglial signaling pathways contribute to central sensitization. In addition to inflammatory mediators, microglia can also influence neuronal plasticity through neurotrophic factors. P2X4R–p38 MAPK signaling promotes the synthesis and release of BDNF from microglia. BDNF can then act on neuronal TrkB signaling and influence TNC neuronal sensitization. In CM models, inhibition of P2X4R or TrkB attenuates pain hypersensitivity and is accompanied by reduced p-ERK and CGRP levels in the TNC (Long et al., 2020). These findings suggest that microglia influence neuronal function not only through inflammatory mediators but also through BDNF-related mechanisms that contribute to central sensitization. Astrocyte-related signaling may also influence trigeminovascular sensitization. The study mentioned above suggests that astrocytes may contribute to central sensitization in CM by regulating glutamate homeostasis (Zhou et al., 2020). At the same time, upon activation of the NF-κB signaling pathway in astrocytes, these cells secrete pro-inflammatory mediators such as IL-1β and TNF-α, which further exacerbate neuroinflammation and lead to the sensitization of pain pathways (Lu et al., 2026). Furthermore, CSD-induced HMGB1 release promotes NF-κB activation in astrocytes, while inhibition of this signaling cascade significantly attenuates CSD-induced trigeminovascular activation (Karatas et al., 2013). Importantly, bidirectional communication between microglia and astrocytes may further modulate these downstream functional effects. However, current mechanistic interpretations are based mainly on evidence from migraine, CM, and other pain models, whereas direct evidence in vestibular migraine remains limited. The role of microglia–astrocyte crosstalk in vestibular migraine and its downstream mechanisms therefore requires further validation in VM-specific models. The above signaling cascades are summarized in Figure 1.

**Figure 1:**
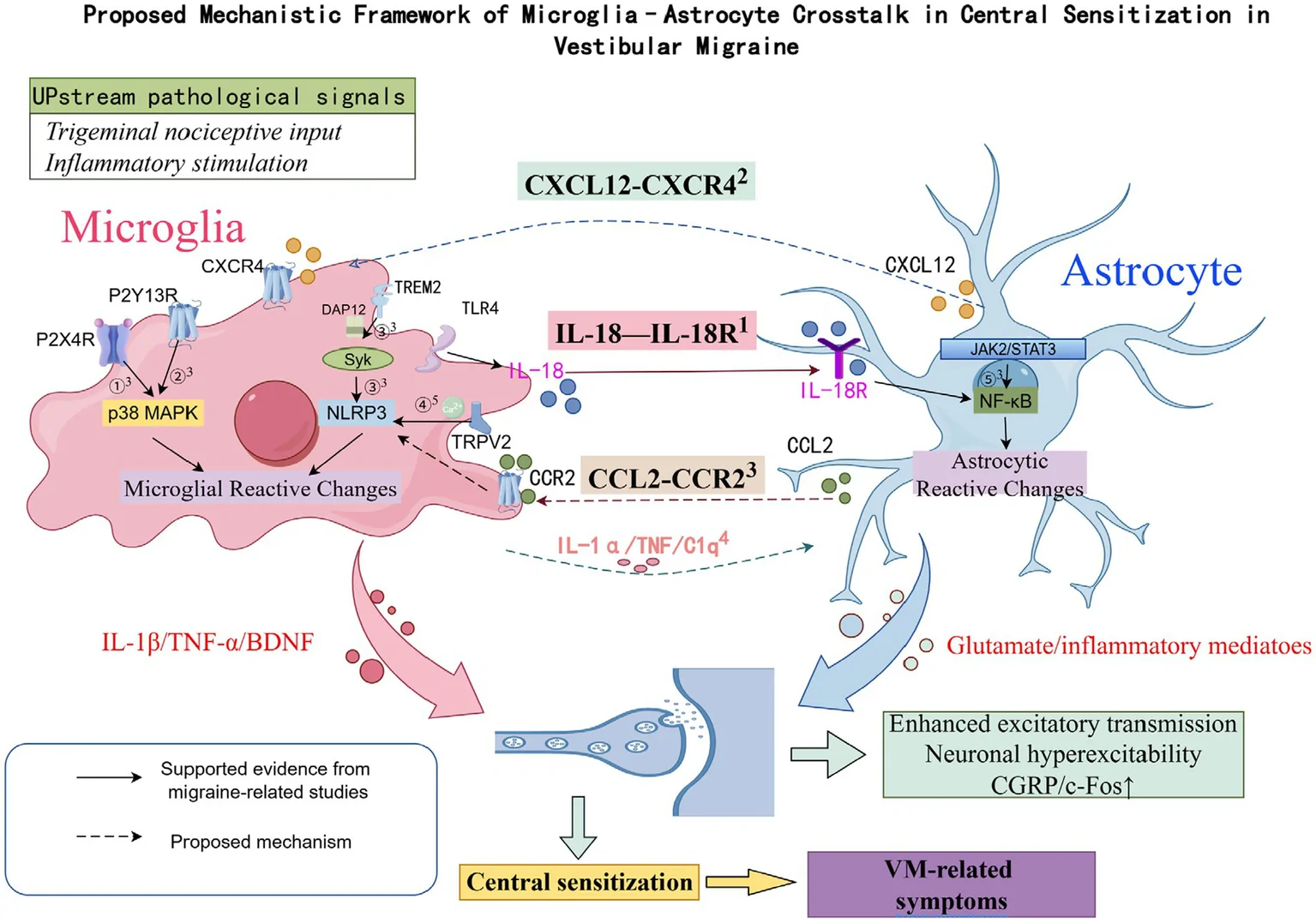
Proposed mechanistic framework of microglia–astrocyte crosstalk in central sensitization in vestibular migraine. Experimental models: 1 Migraine model; 2 Neuropathic pain model; 3 Chronic migraine model; 4 LPS-induced neuroinflammation and CNS injury models; 5 Vestibular migraine model. Intracellular signaling pathways in glial cells: ① P2X4R–p38 MAPK; ② P2Y13R–p38 MAPK; ③ TREM2–SYK–NLRP3; ④ TRPV2-Ca^2+^–NLRP3; ⑤ JAK2/STAT3/NF-κB. By Figdraw.

## 4 Peripheral pathological signals and central sensitization in VM

The process of central sensitization involving glial cell crosstalk between microglia and astrocytes offers a novel theoretical perspective for systematically understanding the pathophysiological mechanisms of VM. However, the upstream mechanisms responsible for this process remain unclear. Increasing evidence suggests that the nervous and immune systems are closely interconnected (Zhang et al., 2024). Peripheral inflammation may contribute to persistent pain sensitization by inducing immune and inflammatory responses in the CNS. In a complete Freund’s adjuvant (CFA)-induced model of peripheral inflammation, Raghavendra et al. (2004) found increased expression of microglial and astrocytic markers in the spinal cord, brainstem, and forebrain, accompanied by sustained increases in the proinflammatory cytokines IL-1β, IL-6, and TNF-α. Notably, the microglial response preceded the astrocytic response. These findings demonstrate that peripheral inflammation can induce widespread glial and inflammatory responses within the CNS. This provides fundamental evidence for the process by which peripheral inflammation may influence glial responses and thereby promote central sensitization (Raghavendra et al., 2004). However, how peripheral inflammatory signals reach the CNS and induce glial responses may involve multiple pathways, with inflammatory mediators and changes in BBB function playing important roles. Research has found that suppression of inflammatory mediators can alleviate VM symptoms or reduce the incidence of VM (Lee et al., 2015). Emerging evidence indicates that interleukin-17A (IL-17A) can penetrate the BBB and enter the medulla oblongata. It activates TNC through microglial cell-mediated neuroinflammation. This mechanism has been demonstrated in a CM model induced by nitroglycerin (Chen et al., 2022). This suggests that abnormal entry of peripheral inflammatory factors across the BBB into the CNS may induce neuroinflammation and represent a critical mechanism in migraine pathogenesis. This mechanism may also provide a pathophysiological basis for VM. The BBB consists of continuous non-fenestrated endothelial cells joined by tight junctions, along with a basement membrane, pericytes, and astrocytic endfeet. At the molecular level, it contains specialized regulatory components, including tight junction proteins, receptors, and transporters that collectively maintain CNS homeostasis (Varatharaj and Galea, 2017). Extensive studies have demonstrated that inflammatory factors such as TNF-α and IL-1β can disrupt tight junction integrity, increase the permeability of cerebral endothelial cells, and ultimately lead to BBB dysfunction (Yang et al., 2016; Zhang et al., 2019). An animal study showed that, in a rat model of chronic trigeminal allodynia induced by repeated dural inflammatory stimulation, increased BBB permeability in the TNC and enhanced trigeminal sensitivity were already present during the episodic stage, while marked microglial and astrocytic responses were mainly observed during the chronic stage. This suggests that, at least in this model, glial responses may not be the earliest pathological event. Instead, they may be gradually recruited during persistent peripheral stimulation and trigeminal activity and contribute to the amplification and maintenance of central sensitization (Fried et al., 2018). Overall, glial cells may be gradually recruited during persistent peripheral inflammation and subsequently contribute to central sensitization.

In addition to the effects of peripheral inflammatory mediators on the CNS through the BBB, migraine-related pathological changes in the audiovestibular periphery may provide another upstream pathway to central sensitization. CGRP is a key mediator in migraine and is also considered an important link between the vestibular system and the inner ear (Demir and Karakus Dilbaz, 2026). During a migraine attack, migraine-related neuropeptides such as CGRP may enter the inner ear microenvironment through a disrupted blood-labyrinth barrier (BLB) and perineural channels (Xu et al., 2026a). An animal study showed that intraperitoneal CGRP injection altered auditory brainstem responses (ABRs) and vestibular sensory evoked potentials (VsEPs) in mice and induced behaviors related to auditory hypersensitivity and postural instability. These changes resemble the phonophobia and postural instability seen in patients with VM, suggesting that CGRP may act, at least in part, on the cochlea and peripheral vestibular structures (Rahman et al., 2025). A recent study showed that repeated NTG injections in a mouse model of CM increased the expression of CGRP and its receptors in cochlear outer hair cells (OHCs). This was accompanied by higher ABR and distortion product otoacoustic emission (DPOAE) thresholds and OHC damage, while CGRP receptor antagonists reduced these changes. More importantly, CGRP may directly affect OHCs by inducing mitochondrial stress and mitochondrial DNA (mtDNA) leakage. This activates Z-DNA-binding protein 1 (ZBP1)-mediated PANoptosis, leading to OHC injury and increased hearing thresholds (Xu et al., 2026b). These findings suggest that the inner ear may be a direct target of migraine-related pathological processes. However, although VM and CM share some auditory and vestibular symptoms, direct evidence for CGRP-mediated inner ear and vestibular pathways comes mainly from CM models. Whether similar changes occur in the vestibular sensory epithelium in VM remains unclear.

Persistent dysfunction or damage of peripheral vestibular sensory structures may alter vestibular input patterns and transmit abnormal sensory signals through the vestibular nerve to the VN and its interconnected trigeminal sensory network, thereby providing sustained sensory input that may contribute to central sensitization. Previous studies have shown that the TNC sends glutamatergic projections to the VN. Thus, abnormal signals from the vestibular and trigeminal systems can be integrated at the central level, supporting a functional connection between the trigeminal and vestibular systems (Zhang et al., 2023b). A recent review on VM also highlighted that trigeminal fibers innervate the labyrinthine vasculature and the inner ear, while CGRP is present in the vestibular sensory epithelium and VN. These findings provide anatomical and physiological support for a functional interaction between the peripheral trigeminal and vestibular systems (Cantillo-Martínez et al., 2025). Importantly, a recent VM animal study found increased TRPV2 expression in TNC microglia, enhanced NLRP3 inflammasome activity, and increased IL-1β signaling following vestibular nerve terminal injury, accompanied by marked central sensitization (Zhai et al., 2025b). These findings suggest that peripheral vestibular abnormalities, central glial responses, and central sensitization may be linked within the same pathological process. Based on the available evidence, we hypothesize that a pathological process involving “peripheral trigger–abnormal sensory input or neuroimmune transmission–glial response–central sensitization” may occur in VM. Peripheral inflammatory mediators may promote microglial and astrocytic inflammatory responses and central sensitization by altering BBB function. Migraine-related neuropeptides may also cause abnormalities in inner ear sensory structures, providing another upstream stimulus to the central trigeminal–vestibular network and glial cells through sustained vestibular input. In this process, glial cells may be gradually recruited during persistent peripheral stimulation and neuronal hyperexcitability and may then amplify neuroinflammation and promote central sensitization through microglia–astrocyte crosstalk.

Taken together, the temporal relationship between glial responses and central sensitization may vary across different pathological conditions. On the one hand, studies of peripheral inflammation and BBB abnormalities suggest that peripheral signals can induce central glial responses and promote central sensitization under specific neuroimmune conditions. This indicates that glial responses may participate in the early development of central sensitization. On the other hand, studies of CSD and the trigeminovascular system suggest that persistent neuronal hyperexcitability or trigeminal sensory input may precede glial responses. In this setting, changes in glial reactivity may occur as a secondary response to abnormal neuronal activity. Thus, peripheral inflammation, local pathological changes in the inner ear, persistent trigeminal sensory input, and sustained neuronal hyperexcitability may all act as upstream signals for glial responses and central sensitization in different pathological contexts. Sustained glial responses and microglia-astrocyte crosstalk may then further increase neuroinflammation and neuronal excitability, forming a bidirectional feedback loop that contributes to the development, amplification, and maintenance of central sensitization.

## 5 Summary

In summary, the pathogenesis of VM is multifactorial. Microglia–astrocyte crosstalk may contribute to central sensitization through inflammatory signaling, trigeminovascular modulation, and dysregulated neurotransmission, thereby promoting disease progression. Peripheral inflammation and inner ear injury may serve as upstream triggers of this process. These findings provide a new perspective for exploring targeted therapeutic strategies for VM. However, in current studies of migraine and VM, evidence for therapies that directly target glial crosstalk remains limited. Most studies instead focus on individual glial cell populations or on suppressing neuroinflammation. Despite these advances, several important limitations should be acknowledged. Most mechanistic insights are derived from animal models, and their direct relevance to human VM remains uncertain. Moreover, the lack of human-based evidence substantially limits our understanding of how these mechanisms translate into clinical manifestations. Bridging the gap between experimental findings and clinical application therefore remains a major challenge. Future studies should prioritize human-centered approaches, including neuroimaging, cerebrospinal fluid analysis, and single-cell technologies, to validate glial reactivity patterns in patients with VM. Advances in high-throughput techniques, such as single-cell sequencing and spatial transcriptomics, may further enable precise characterization of glial heterogeneity and facilitate the identification of novel biomarkers and therapeutic targets. In addition, the development of more representative animal models that recapitulate both headache-like behaviors and vestibular dysfunction will be critical for dissecting stage-specific mechanisms of central sensitization. Future research should also explore targeted pharmacological and non-pharmacological interventions aimed at modulating glial reactivity and suppressing key inflammatory pathways. Such strategies may ultimately provide new therapeutic avenues for the management of VM.

## Glossary

ABR: Auditory brainstem response
ATP: Adenosine triphosphate
BBB: Blood–brain barrier
BDNF: Brain-derived neurotrophic factor
CCL2: C-C motif chemokine ligand 2
CGRP: Calcitonin gene-related peptide
CM: Chronic migraine
CNS: Central nervous system
CSD: Cortical spreading depolarization
CXCL10: C-X-C motif chemokine ligand 10
CXCL12: C-X-C motif chemokine ligand 12
CXCR3: C-X-C motif chemokine receptor 3
CXCR4: C-X-C motif chemokine receptor 4
EAAT2: Excitatory amino acid transporter 2
ERK: Extracellular signal-regulated kinase
GFAP: Glial fibrillary acidic protein
HMGB1: High-mobility group box 1
IL-1β: Interleukin-1β
IL-6: Interleukin-6
IL-18: Interleukin-18
IL-18R: Interleukin-18 receptor
IS: Inflammatory soup
LPS: Lipopolysaccharide
NF-κB: Nuclear factor kappa B
NMDA: N-methyl-D-aspartate
NTG: Nitroglycerin
OHC: Outer hair cell
P2X4R: P2X4 receptor
P2Y13R: P2Y13 receptor
p38 MAPK: p38 mitogen-activated protein kinase
p-ERK: Phosphorylated extracellular signal-regulated kinase
SYK: Spleen tyrosine kinase
TLR4: Toll-like receptor 4
TNC: Trigeminal nucleus caudalis
TNF-α: Tumor necrosis factor-alpha
TREM2: Triggering receptor expressed on myeloid cells 2
TRPV2: Transient receptor potential vanilloid 2
VM: Vestibular migraine
VN: Vestibular nucleus
XZDL: Xiongzhi Dilong decoction
α7nAChR: α7 nicotinic acetylcholine receptor
